# Utility of the Endoscopic Pressure Study Integrated System in Identifying Silent Gastroesophageal Reflux Disease During Routine Health Check‐ups

**DOI:** 10.1002/deo2.70204

**Published:** 2025-09-08

**Authors:** Yohei Nishikawa, Haruhiro Inoue, Kazuki Yamamoto, Tomona Sakurai, Yukiko Okada, Kei Ushikubo, Kohei Shigeta, Ippei Tanaka, Satoshi Abiko, Mayo Tanabe, Takayoshi Ito, Noboru Yokoyama, Naoyuki Uragami

**Affiliations:** ^1^ Digestive Diseases Center Showa Medical University Koto Toyosu Hospital Tokyo Japan; ^2^ Preventive Medicine Center Showa Medical University Toyosu Clinic Tokyo Japan

**Keywords:** endoscopic pressure study integrated system, functional endoscopy, gastroesophageal reflux disease, health check‐ups, lower esophageal sphincter

## Abstract

**Objectives:**

The endoscopic pressure study integrated system (EPSIS) is a novel functional endoscopic modality that records intragastric pressure (IGP) waveforms during CO_2_ insufflation to evaluate lower esophageal sphincter (LES) function and diagnose gastroesophageal reflux disease (GERD). Although previous studies have applied EPSIS to symptomatic patients, its utility in asymptomatic individuals remains unclear. This study aimed to evaluate the diagnostic value of EPSIS in detecting silent GERD—defined as asymptomatic erosive esophagitis—and associated risk factors in individuals undergoing routine health check‐ups.

**Methods:**

We retrospectively analyzed 185 participants who underwent esophagogastroduodenoscopy (EGD) and EPSIS as part of routine health check‐ups at a single center between November 2024 and March 2025. Participants were classified into erosive and non‐erosive groups based on the presence or absence of mucosal breaks (Grade A or higher) according to the Los Angeles classification. Background characteristics, EPSIS parameters, and endoscopic findings were compared.

**Results:**

EPSIS was safely performed in all participants without adverse events. The erosive esophagitis group showed significantly more flat waveform patterns (39.4% vs. 14.5%, *p* = 0.002) and lower maximum IGP values (16.7 mmHg vs. 18.3 mmHg, *p* = 0.008) compared to the non‐erosive group.

**Conclusion:**

EPSIS enables a safe and objective assessment of LES function and may support the identification of erosive esophagitis in asymptomatic individuals. It may hold promise as a functional diagnostic tool for the detection of silent GERD and may support preventive strategies during routine endoscopy.

## 1 Introduction

The endoscopic pressure study integrated system (EPSIS) is a simple and effective endoscopic tool designed to evaluate the function of the lower esophageal sphincter (LES) [1]. During esophagogastroduodenoscopy (EGD), the LES competency can be simply assessed by intragastric CO_2_ insufflation while observing the cardia in a retroflexed view from the stomach. CO_2_ insufflation activates the LES, and its competency is visually recognized as the “scope‐holding sign” [2, 3]. Specifically, the LES activation acts as an anti‐reflux valve, retaining CO_2_ gas within the stomach during insufflation. Upon reaching a certain intragastric pressure (IGP) threshold, the LES relaxes, releasing the accumulated CO_2_ gas as a belch. EPSIS measures the IGP as a pressure waveform during the process of insufflation, providing valuable insights into LES functionality.

EPSIS has demonstrated diagnostic utility in detecting gastroesophageal reflux disease (GERD), with previous studies indicating a significant correlation between EPSIS parameters and 24‐h pH‐impedance monitoring results [1]. Specifically, GERD patients often exhibit flat waveform patterns and lower maximum IGP values during EPSIS assessments. Additionally, it has been reported that EPSIS findings are associated with endoscopic manifestations such as erosive esophagitis and Barrett's esophagus [4]. These observations suggest that EPSIS serves as a valuable adjunct to existing diagnostic modalities for GERD in routine clinical practice.

Previous studies have primarily applied the EPSIS to patients with confirmed GERD or those exhibiting GERD symptoms necessitating further diagnostic evaluations, such as 24‐h pH‐impedance monitoring [1, 4, 5]. These investigations have focused on symptomatic individuals, often categorizing patients with functional heartburn within the EPSIS‐negative group. In contrast, the present study utilized EPSIS on asymptomatic participants undergoing routine health check‐up endoscopy. Our objective was to assess the diagnostic utility of EPSIS in detecting silent GERD—defined as asymptomatic erosive esophagitis—and identifying associated risk factors. Furthermore, this evaluation aimed to establish new diagnostic criteria for EPSIS parameters in a healthy, symptom‐free cohort, including the determination of appropriate cutoff values. This study aimed to provide evidence supporting the role of EPSIS in preventive strategies for GERD and to highlight its potential to enhance diagnostic accuracy in clinical settings.

## 2 Materials and Methods

This retrospective cohort study analyzed data collected at Showa Medical University Koto Toyosu Hospital, Tokyo, Japan, from November 2024 to March 2025. Participants underwent routine health check‐ups at the Preventive Medicine Center of Showa Medical University Toyosu Clinic and subsequently underwent EGD followed by the EPSIS procedure at the Digestive Diseases Center of Showa Medical University Koto Toyosu Hospital. Only those who provided written informed consent for participation and for the EPSIS procedure were included in the study. This study protocol was approved by the Ethics Committee of Showa Medical University Koto Toyosu Hospital (IRB Registration Number: 2025‐0108). All procedures adhered to the guidelines outlined in the Declaration of Helsinki. Written informed consent was obtained from all participants.

All participants were considered asymptomatic, defined as individuals who did not have GERD symptoms warranting medical evaluation or treatment, and who therefore underwent voluntary screening endoscopy as part of a medical check‐up at their own expense without using medical insurance. Individuals receiving medication, including proton pump inhibitors (PPIs) or other treatments for gastrointestinal diseases, were excluded. Additional exclusion criteria encompassed age under 18 years, pregnancy, the presence of complex comorbidities, and general contraindications for endoscopic procedures.

Background information, including age, gender, body mass index (BMI), smoking history, and alcohol consumption, was collected. Endoscopic findings such as erosive esophagitis, hernia, and atrophic gastritis were documented. Participants were classified into two groups: those with erosive esophagitis and those without erosive esophagitis, based on the presence or absence of mucosal breaks of Grade A or higher according to the Los Angeles (LA) classification. Background information, endoscopic findings, and EPSIS parameters were then compared between these groups. Receiver operating characteristic (ROC) curve analyses of EPSIS parameters were conducted to determine the diagnostic accuracy with the optimal cutoff values for differentiating erosive esophagitis from the non‐erosive group.

### 2.1 EPSIS Procedure

**FIGURE 1:**
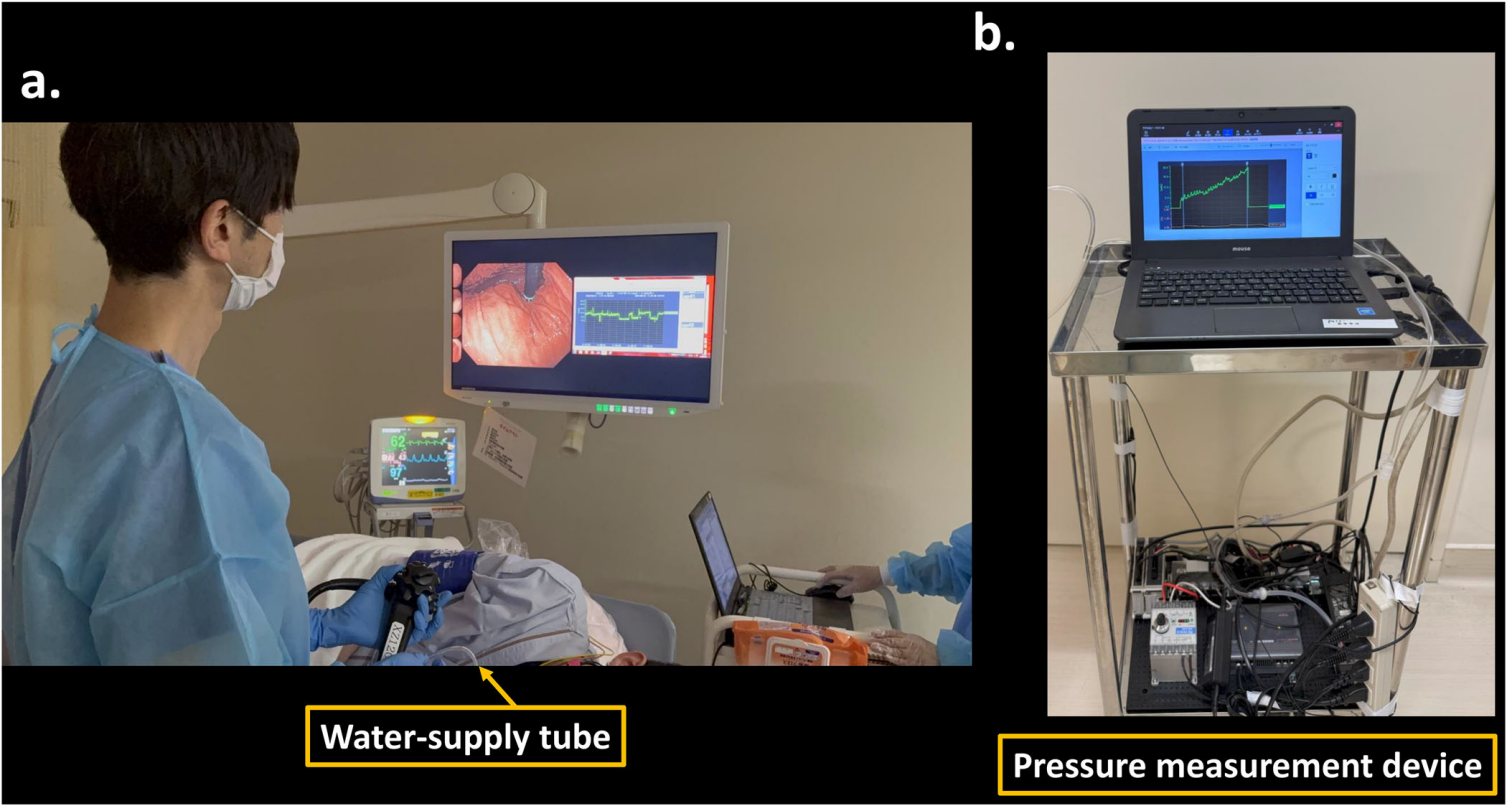
Endoscopic pressure study integrated system (EPSIS) Procedure. (a) EPSIS was performed under sedation with propofol, using a multiparameter monitor to continuously observe vital signs, including electrocardiogram, oxygen saturation (SpO_2_), and blood pressure. A water‐supply tube was connected to the working channel of the endoscope. Intragastric CO_2_ insufflation was administered while intragastric pressure (IGP) waveforms were recorded under direct endoscopic visualization of the cardia on the same monitor. (b) The other end of the tube was connected to a pressure measurement device, and IGP during insufflation was recorded as a pressure waveform.

EPSIS was performed using a high‐definition endoscope (GIF‐XZ1200; Olympus Corp., Tokyo, Japan) under sedation with propofol, with continuous monitoring of vital signs, including electrocardiogram, oxygen saturation (SpO_2_), and blood pressure (Figure 1a). After the examination, patients were observed for an additional 15 min in the same manner.

Following a routine screening endoscopy, a water‐supply tube (AF‐WT; Forte Grow Medical Corp., Tochigi, Japan) was connected to the working channel of the endoscope (Figure 1a). The other end of the tube was attached to a pressure measurement device (TR‐W550, TR‐TH08, and AP‐C35; Keyence, Osaka, Japan) (Figure 1b). Continuous insufflation was provided while measuring IGP waveforms under direct endoscopic observation of the cardia (Figure 1a). To enhance safety, a CO_2_ insufflator (UCR; Olympus Corp.) with a high‐flow tube (MAJ‐1741; Olympus Corp.) was used for insufflation, maintaining a steady flow rate of approximately 1.3 L/min. Insufflation was stopped when the cardia opened and belching occurred. The IGP waveforms were recorded during this process.

To avoid Mallory‐Weiss tears (MWTs), the maximum IGP was limited to 25 mmHg, and immediate deflation was performed when this threshold was reached. If the mucosa showed minute petechial hemorrhages as endoscopic findings, which might be precursors to MWTs, insufflation was also immediately stopped.

### 2.2 Evaluation of EPSIS Parameters

**FIGURE 2:**
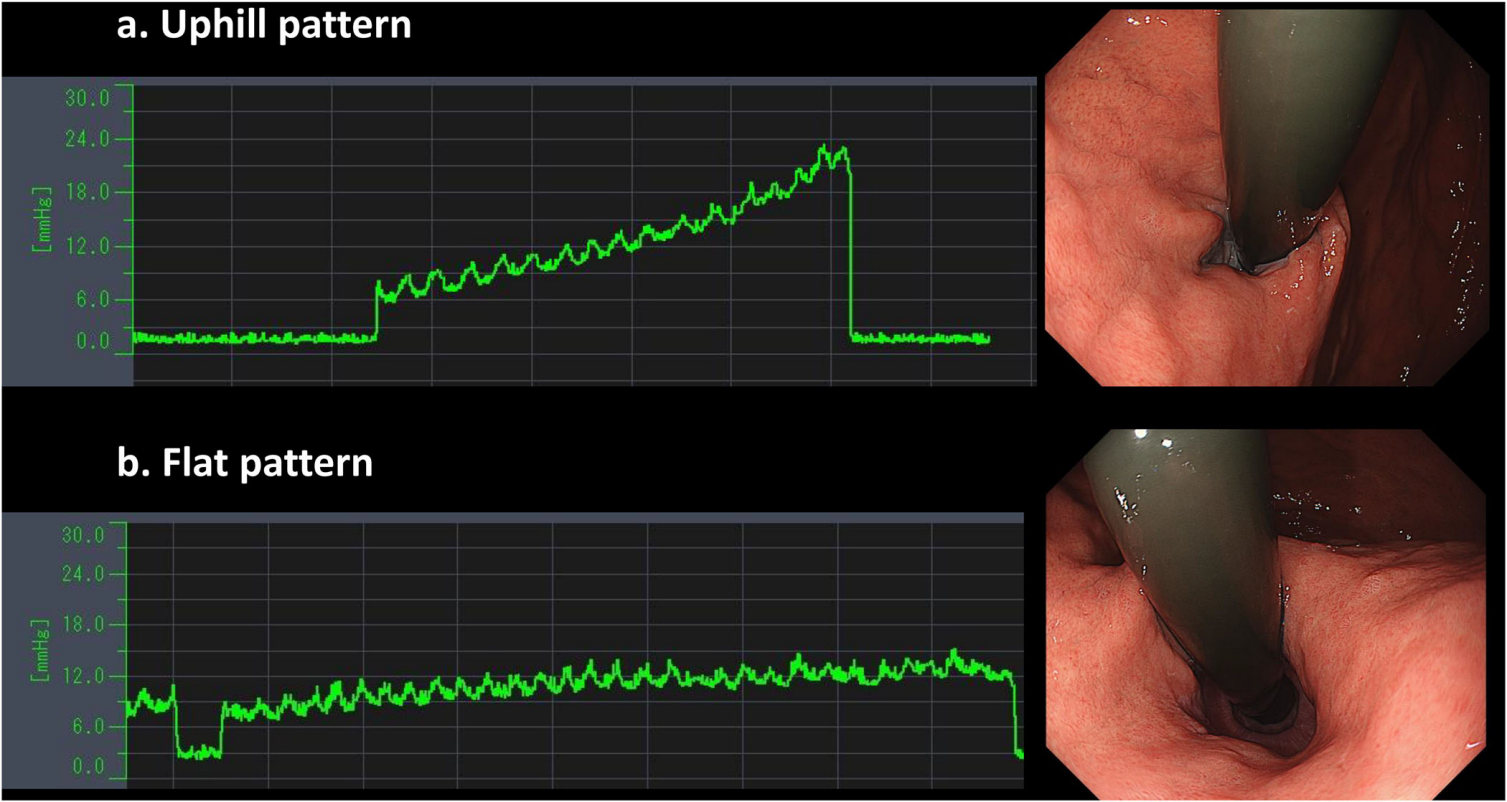
Endoscopic pressure study integrated system (EPSIS) Waveform Patterns (Uphill vs. Flat). (a) The uphill pattern is characterized by a continuous increase in intragastric pressure (IGP) during insufflation. The endoscopic finding is a scope‐holding sign positive, indicating that the endoscope holds the lower esophageal sphincter (LES) and does not release it. (b) The flat pattern is characterized by only a slight increase in IGP with sustained belching during insufflation. The endoscopic finding is scope‐holding sign negative, indicating that the endoscope does not hold the LES.

**FIGURE 3:**
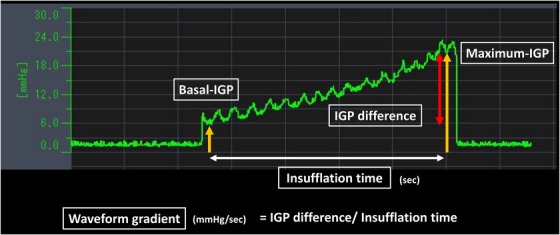
Endoscopic pressure study integrated system (EPSIS) Parameters. Basal IGP (mmHg) refers to the intragastric pressure at the start of CO_2_ insufflation. Maximum IGP (mmHg) is defined as the intragastric pressure at the time of belching or when the upper IGP limit of 25 mmHg is reached during continuous CO_2_ insufflation. The IGP difference (mmHg) is calculated as the difference between the maximum and basal IGP. The waveform gradient (mmHg/second) is obtained by dividing the IGP difference by the insufflation time.

EPSIS waveforms were classified into two main patterns (uphill and flat) (Figure 2). The waveform classification was independently assessed by two board‐certified endoscopists of the Japan Gastroenterological Endoscopy Society, each of whom had performed over 100 EPSIS examinations. The following waveform parameters were recorded: basal IGP, maximum IGP, and insufflation time. Based on these parameters, the IGP difference and the waveform gradient were calculated. The IGP difference was calculated as the difference between the maximum IGP and basal IGP. The waveform gradient was defined as the IGP difference divided by the insufflation time (Figure 3).

### 2.3 Endoscopic Assessment of Hernia and Atrophic Gastritis

Endoscopic assessment of hernia was based on two parameters: cardiac opening (CO) and sliding hernia (SH). CO was defined as the diameter of the cardia opening (cm), and SH was defined as the distance from the diaphragmatic crus to the squamocolumnar junction (cm). In accordance with previous studies, participants with a CO greater than 3 cm and an SH greater than 2 cm were classified as having an endoscopically significant hernia [6].

Atrophic gastritis was defined as significant mucosal atrophy corresponding to C‐II or higher, based on the Kimura‐Takemoto classification [7].

### 2.4 Statistical Analysis

Continuous variables were summarized as means with standard deviations (SD), while categorical variables were presented as counts with corresponding percentages. Comparisons of continuous variables between groups were conducted using Student's *t*‐test, and categorical variables were analyzed using Fisher's exact test. Statistical significance was defined as a *p*‐value less than 0.05. To determine the diagnostic accuracy with the optimal cut‐off values for EPSIS parameters, Youden's J statistic was employed. Logistic regression analysis was performed to identify predictors of erosive esophagitis. Variables with a *p*‐value less than 0.05 in the univariate analysis were included in the multivariate model. All statistical analyses were conducted using JMP software (SAS Institute Japan Ltd., Tokyo, Japan).

## 3 Results

### 3.1 Participants’ Demographics and Endoscopic Findings

A total of 185 participants were enrolled in the study, with a mean age of 47.6 years (SD: 8.8). Table 1 summarizes the participants' demographic characteristics and endoscopic findings related to erosive esophagitis. According to the LA classification, 152 participants (82.2%) had no erosive esophagitis or Grade M, 27 (14.6%) had Grade A, 5 (2.7%) had Grade B, 1 (0.5%) had Grade C, and none (0%) had Grade D esophagitis.

**TABLE 1:** Basic information and endoscopic findings (*n* = 185) of the participants.

**Basic information**	
Age, years, mean ± SD	47.6 ± 8.8
Male sex, *n* (%)	111 (60)
BMI, kg/m^2^, mean ± SD	22.9 (3.7)
Current smoker, *n* (%)	32 (17.3)
Alcohol (heavy drinker), *n* (%)	41 (22.2)
**Endoscopic findings**	
Sliding hernia >2 cm, *n* (%)	5 (2.7)
Cardiac opening >3 cm, *n* (%)	5 (2.7)
Atrophic gastritis ≥ C‐II, *n* (%)	29 (15.7)
**Erosive esophagitis (LA classification)**	
None or Grade M, *n* (%)	152 (82.2)
Grade A, *n* (%)	27 (14.6)
Grade B, *n* (%)	5 (2.7)
Grade C, *n* (%)	1 (0.5)
Grade D, *n* (%)	0 (0)

Abbreviations: BMI, body mass index; LA, Los Angeles; SD, standard deviation.

### 3.2 Clinical Characteristics and Endoscopic Findings of Participants With and Without Erosive Esophagitis

Basic demographic and endoscopic findings were compared between 33 participants in the erosive esophagitis group and 152 participants in the non‐erosive group (Table 2). The erosive esophagitis group had a significantly higher proportion of males (90.9% vs. 53.5%, *p* < 0.001) and a higher mean BMI (25.4 vs. 22.3, *p* < 0.001) compared to the non‐erosive group. Additionally, the prevalence of current smokers was significantly higher in the erosive esophagitis group (30.3% vs. 14.5%, *p* = 0.041).

**TABLE 2:** Clinical characteristics of participants with/without erosive esophagitis.

	Erosive (+) (*n* = 33)	Erosive (‐) (*n* = 152)	*p*‐Value
Age, years, mean ± SD	48.4 (8.5)	47.4 (8.9)	0.562
Male sex, *n* (%)	30 (90.9)	81 (53.3)	< 0.001
BMI, kg/m^2^, mean ± SD	25.4 (4.6)	22.3 (3.3)	< 0.001
Current smoker, *n* (%)	10 (30.3)	22 (14.5)	0.041
Alcohol (heavy drinker), *n* (%)	9 (27.3)	32 (21.1)	0.489
Endoscopic findings			
Sliding hernia >2 cm, *n* (%)	3 (9.1)	2 (1.3)	0.040
Cardiac opening >3 cm, *n* (%)	1 (3.0)	4 (2.6)	1.000
Atrophic gastritis ≥ C‐II, *n* (%)	5 (15.2)	24 (15.8)	1.000

Abbreviations: GERD, gastroesophageal reflux disease; SD, standard deviation.

A heavy drinker was defined as an individual consuming more than 40 grams of pure alcohol per day.

Among the endoscopic findings, SH was more frequently observed in the erosive esophagitis group than in the non‐erosive group (9.1% vs. 1.3%, *p* = 0.040).

### 3.3 Comparison of EPSIS Parameters With and Without Erosive Esophagitis

EPSIS procedures were successfully performed on all participants without any adverse events. The EPSIS parameters revealed a significantly higher prevalence of flat waveform patterns in the erosive esophagitis group compared to the non‐erosive group (39.4% vs. 14.5%, *p* = 0.002). The maximum IGP was significantly lower in the erosive esophagitis group than in the non‐erosive group (16.7 mmHg vs. 18.3 mmHg, *p* = 0.008). Similarly, the IGP difference (8.7 mmHg vs. 10.9 mmHg, *p* = 0.002) and waveform gradient (0.185 mmHg/sec vs. 0.229 mmHg/sec, *p* = 0.049) were significantly reduced in the erosive esophagitis group. A comprehensive summary of the EPSIS parameters is presented in Table 3.

**TABLE 3:** Comparison of endoscopic pressure study integrated system (EPSIS) parameters with/without erosive esophagitis.

	Erosive (+) (*n* = 33)	Erosive (‐) (*n* = 152)	*p*‐Value
Flat pattern, *n* (%)	13 (39.4)	22 (14.5)	0.002
Maximum IGP, mmHg, mean ± SD	16.7 ± 3.4	18.3 ± 3.2	0.008
IGP difference, mmHg, mean ± SD	8.7 ± 3.6	10.9 ± 3.5	0.002
Waveform gradient, mmHg/s, mean ± SD	0.185 ± 0.125	0.229 ± 0.116	0.049

Abbreviations: EPSIS, endoscopic pressure study integrated system; IGP, intragastric pressure; SD, standard deviation.

ROC analyses were conducted to evaluate the diagnostic performance of each EPSIS parameter in identifying erosive esophagitis. Among these, the IGP difference yielded the largest area under the curve (AUC) value, with an optimal cutoff value of 10.8 mmHg (sensitivity: 75.8%, specificity: 53.3%, positive predictive value [PPV]: 26.0%, negative predictive value [NPV]: 81.8%, AUC: 0.667). The maximum IGP and waveform gradient had AUC values of 0.638 (cutoff: 16.8 mmHg; sensitivity: 45.5%, specificity: 75.7%, PPV: 28.8%, NPV: 86.5%) and 0.662 (cutoff: 0.197 mmHg/sec; sensitivity: 75.8%, specificity: 57.2%, PPV: 27.8%, NPV: 91.6%), respectively (Table 4).

**TABLE 4:** Diagnostic ability of endoscopic pressure study integrated system (EPSIS) parameters for erosive esophagitis.

	Optimal cutoff	AUROC	Sensitivity (%)	Specificity (%)	PPV (%)	NPV (%)
Maximum IGP	16.8	0.638	45.5	75.7	28.8	86.5
IGP difference	10.8	0.667	75.8	53.3	26.0	81.8
Waveform gradient	0.197	0.662	75.8	57.2	27.8	91.6

Abbreviations: AUROC, area under the receiver operating characteristic curve; EPSIS, endoscopic pressure study integrated system; IGP, intragastric pressure; NPV, negative predictive value; PPV, positive predictive value.

### 3.4 Multivariate Analysis for the Risk Factors of Erosive Esophagitis

Multivariate analysis identified male sex (odds ratio [OR] = 5.65, p = 0.013) and higher BMI (OR = 1.17, p = 0.016) as independent predictors of erosive esophagitis. (Table 5).

**TABLE 5:** Univariate and multivariate analysis of clinical characteristics and endoscopic pressure study integrated system (EPSIS) parameters with/without erosive esophagitis.

	Erosive esophagitis		Univariate analysis	Multivariate analysis	
	Erosive (+) (*n* = 33)	Erosive (‐) (*n* = 152)	*p*‐Value	Adjusted odds ratio (95%CI)	*p*‐Value
Male sex, *n* (%)	30 (90.9)	81 (53.3)	<0.001	5.65 (1.44–22.03)	0.013
BMI, kg/m^2^, mean ± SD	25.4 (4.6)	22.3 (3.3)	<0.001	1.17 (1.03–1.32)	0.016
Current smoker, *n* (%)	10 (30.3)	22 (14.5)	0.041	1.24 (0.46–3.35)	0.670
Flat pattern, *n* (%)	13 (39.4)	22 (14.5)	0.002	2.49 (0.62–10.01)	0.200
Maximum IGP, mmHg, mean ± SD	16.7 ± 3.4	18.3 ± 3.2	0.008	0.969 (0.79–1.18)	0.758
IGP difference, mmHg, mean ± SD	8.7 ± 3.6	10.9 ± 3.5	0.002	0.952 (0.80–1.13)	0.577
Waveform gradient, mmHg/s, mean ± SD	0.185 ± 0.125	0.229 ± 0.116	0.049	4.99 (0.06–395.34)	0.471

Abbreviations: BMI, body mass index; IGP, intragastric pressure; SD, standard deviation.

## 4 Discussion

This study demonstrated significant differences in all EPSIS parameters between asymptomatic participants with erosive and non‐erosive esophagitis during routine health check‐ups. EPSIS is a novel measurement device capable of evaluating LES function, which is a key factor in the pathogenesis of GERD [8, 9, 10]. These results highlight the diagnostic value and potential clinical utility of EPSIS through objective assessment based on established endoscopic GERD diagnoses. Importantly, all EPSIS measurements were performed safely without any adverse events, reinforcing its utility as a functional endoscopic diagnostic tool for GERD in clinical practice.

This study highlights two potential clinical benefits of incorporating EPSIS into health check‐ups. First, EPSIS is expected to facilitate the early detection and risk assessment of GERD by providing a simple and objective measurement of LES function. In this study, to evaluate the more sensitive diagnostic capability of EPSIS, the erosive esophagitis group was defined as LA grade A or higher, representing minor mucosal damage that can often be managed with lifestyle modification alone. Importantly, EPSIS parameters were able to detect such minor mucosal changes, even in asymptomatic individuals. Early detection of such subtle abnormalities could support timely lifestyle interventions before symptom onset. In Asia, GERD prevalence is increasing due to westernized diets and rising obesity rates [11, 12]. This trend is associated with a growing incidence of Barrett's esophagus and adenocarcinoma [13, 14], making the identification of risk factors essential. In this study, male sex, higher BMI, and smoking were more common in the erosive esophagitis group, and multivariate analysis identified male sex and elevated BMI as independent risk factors. These findings are consistent with previous research [12, 15, 16]. Individuals with these risk factors should be encouraged to undergo early detection of GERD and to make lifestyle modifications—such as weight loss, smoking cessation, regular exercise, and a healthy diet—which have been shown to reduce GERD risk [17, 18, 19]. Second, EPSIS offers the possibility of objectively evaluating LES function over time after detection. It may be particularly useful for monitoring changes following lifestyle interventions. Currently, objective methods for evaluating the effectiveness of such interventions are largely limited to endoscopy and 24‐h pH‐impedance monitoring [20, 21, 22]. EPSIS provides a minimally invasive alternative that can be repeated periodically to monitor LES function and reflux dynamics over time. For example, individuals with high BMI and flat EPSIS waveform patterns could undergo follow‐up endoscopy combined with EPSIS to evaluate whether lifestyle changes have improved LES function and reduced GERD risk. Future studies should examine longitudinal changes in EPSIS parameters among high‐risk individuals to determine their prognostic value and refine preventive strategies.

**FIGURE 4:**
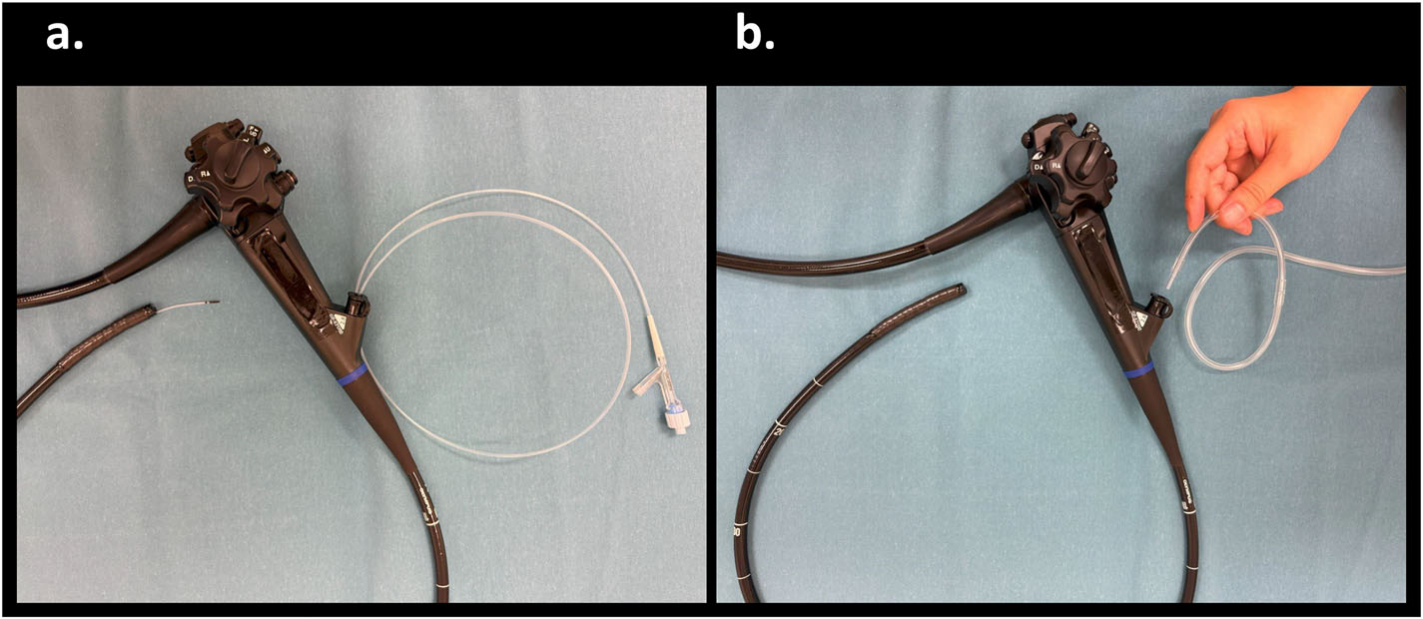
Original endoscopic pressure study integrated system (EPSIS) versus updated EPSIS. (a) The original EPSIS method was performed using an intragastric catheter. A through‐the‐scope catheter was connected to an internal pressure measurement device. (b) In the updated EPSIS method, a water‐supply tube was directly connected to the endoscope's working channel, eliminating the need for a through‐the‐scope catheter.

While this study has research significance, EPSIS has so far been primarily applied to populations with severe GERD symptoms requiring 24‐h pH monitoring. By evaluating EPSIS in a population with largely normal LES function and establishing cutoff values, our findings may contribute to the future development and broader clinical application of EPSIS as a diagnostic tool. Previous studies have identified a flat waveform pattern and a low maximum IGP of 18.7 mmHg as potential predictors for GERD [1]. Additionally, the IGP difference and waveform gradient have shown diagnostic utility in GERD groups classified by 24‐h pH‐impedance monitoring, particularly when the acid exposure time exceeds 6% [5]. The results of the current study support these earlier findings. Participants with erosive esophagitis exhibited a flat waveform and lower maximum IGP values, with a newly identified cutoff of 16.8 mmHg. The IGP difference and waveform gradient had AUROC values of 0.667 and 0.662, respectively, suggesting that decreases in these parameters may have moderate diagnostic value for identifying erosive esophagitis in an asymptomatic screening population. The newly identified cutoff value of 16.8 mmHg for maximum IGP in this study is slightly lower than the 18.7 mmHg reported in the initial EPSIS report. The previously reported cutoff value of 18.7 mmHg was established in a symptomatic GERD population—specifically, among patients with significant symptoms requiring 24‐h pH monitoring—using LA grade A or higher esophagitis as the reference standard, which is the same criterion applied in the present study. Differences between symptomatic and asymptomatic groups may partly account for the lower value observed. In addition, methodological advancements in EPSIS—particularly the development of a catheter‐free technique—likely contributed to the lower cutoff value observed. This innovation has simplified the procedure and improved safety by eliminating the need to introduce foreign materials into the body [23]. The variation in IGP values is likely influenced by differences in the diameter of the measurement pathway between the traditional catheter‐based and catheter‐free methods (Figure 4). However, previous reports have demonstrated that the updated EPSIS retains comparable diagnostic performance to the original version [23]. The cutoff value established in this asymptomatic population may serve as a potential risk indicator for silent GERD in health check‐up settings. To establish a diagnostic cutoff for GERD that is applicable across both symptomatic and asymptomatic cohorts, further large‐scale studies will be necessary. We consider this study a preliminary step toward that goal.

This study has several limitations. First, it was conducted at a single center with a relatively limited sample size, which may affect the generalizability of the findings. To establish more accurate cutoff values and improve robustness, multicenter data collection is already underway. Second, the study population consisted of participants undergoing endoscopy as part of uninsured health check‐ups, representing an asymptomatic and ostensibly healthy group. Therefore, a detailed assessment of clinical symptoms was not feasible, and some participants with subclinical symptoms not warranting medical attention may have been included. Nonetheless, this cohort is clearly distinct from those in previous studies, which primarily focused on patients with confirmed GERD or overt GERD symptoms. Third, the endoscopists performed the EPSIS procedures after diagnosing GERD based on the LA classification. This overlap in roles may have introduced diagnostic bias, as knowledge of the endoscopic diagnosis could have influenced the interpretation of the EPSIS results. To minimize this risk, EPSIS parameters were analyzed after the examination, and waveform classification (uphill or flat) was independently determined by two endoscopists, as mentioned above. Finally, in the multivariate analysis, EPSIS parameters did not emerge as independent predictors of erosive esophagitis. However, this finding does not negate their diagnostic utility, as the univariate analysis demonstrated significant associations. Several factors may explain the lack of statistical independence in the multivariate model. EPSIS parameters are likely correlated with established GERD risk factors such as BMI and sex, which may have attenuated their significance when included together. In addition, the relatively small number of erosive esophagitis cases may have reduced the statistical power to detect independent effects. Further studies involving larger and more diverse populations are warranted to determine whether EPSIS parameters can serve as independent predictors and to explore their integration into multivariable risk prediction models.

In conclusion, this study demonstrated that EPSIS may serve as a valuable tool for detecting silent GERD, defined as asymptomatic erosive esophagitis. These findings underscore the potential of EPSIS not only in routine clinical practice but also in the field of preventive medicine. Broader implementation could facilitate early detection and intervention, ultimately reducing the burden of GERD. Future efforts should aim to establish EPSIS as a standard functional endoscopic modality for both screening and prevention in symptomatic and asymptomatic populations.

## Conflicts of Interest

Dr. Haruhiro Inoue serves as an advisor to Olympus Corporation. He received educational grants from Olympus Corporation. All other authors declare no conflicts of interest. This relationship had no influence on the study design, data collection, analysis, or interpretation.

## Ethics Statement

This study was approved by the Showa Medical University Hospital Research Ethics Committee (institutional review board registration number: 2025‐0108). Informed consent was obtained from all patients prior to undergoing EPSIS.

## Clinical Trial Registration

N/A.
